# TEMPORAL COMPARISON OF WHEEZING PREVALENCE IN THE FIRST YEAR OF LIFE IN SÃO PAULO: INTERNATIONAL STUDY OF WHEEZING IN INFANTS

**DOI:** 10.1590/1984-0462/;2018;36;4;00016

**Published:** 2018

**Authors:** Carolina Sanchez Aranda, Gustavo Falbo Wandalsen, Ana Caroline Cavalcanti Dela Bianca, Ellen de Oliveira Dantas, Javier Mallol, Dirceu Solé

**Affiliations:** a Universidade Federal de São Paulo, São Paulo, SP, Brasil.; b Universidade de Santiago, Santiago, Chile.

**Keywords:** Infant, Respiratory sounds/wheezing, Prevalence;Asthma, Lactente, Sons respiratórios, Prevalência, Asma

## Abstract

**Objective:**

To assess the prevalence and severity of wheezing in the first year of life of infants, using the standardized protocol of the *Estudio Internacional de Sibilancias en Lactantes*- phase 3, and compare the values obtained with those found in phase 1, conducted at the same center.

**Methods:**

Between 2009 and 2010, parents and guardians of infants answered the written questionnaire of the *Estudio Internacional de Sibilancias en Lactantes* - phase 3, and its results were compared to those of phase 1, performed between 2005 and 2006. We divided the infants into wheezing and non-wheezing. The wheezing group was stratified according to the frequency of episodes: occasional wheezing - less than three -, and recurrent wheezing - three or more.

**Results:**

Wheezing prevalence was similar in both phases (44.6 versus 46%). Regarding frequency, the prevalence of occasional wheezing increased (19.4 versus 23%; p=0.03) and recurrent wheezing decreased (26.7 versus 21.6%; p=0.005). Also, diagnosis of asthma (7.5 versus 21.8%), use of inhaled corticosteroids (11.7 versus35%), and hospitalization for wheezing (19.7 versus 32.6%) grew significantly in phase 3. This period coincides with the Influenza A (H1N1) pandemic, which could have contributed to this outcome.

**Conclusions:**

Wheezing prevalence in the first year of life remains high. Despite the temporal assessment showing a decrease in the prevalence of recurrent wheezing, a significant increase in its morbidity was identified due to the higher number of hospitalizations. In addition, there were signs of improvement in the wheezing management of infants, reflected by an increase in the diagnosis of asthma and a greater indication of preventive treatments.

## RESUMO

**Objetivo:**

Avaliar a prevalência e a gravidade da sibilância em lactentes no primeiro ano de vida, utilizando o protocolo padronizado do *Estudio Internacional de Sibilancias en Lactantes*- fase 3, e comparar os valores obtidos com os observados no *Estudio Internacional de Sibilancias en Lactantes*- fase 1, realizado no mesmo centro.

**Métodos:**

Entre 2009 e 2010, pais e responsáveis de lactentes responderam ao questionário escrito do *Estudio Internacional de Sibilancias en Lactantes*- fase 3, e os resultados obtidos foram comparados aos do *Estudio Internacional de Sibilancias en Lactantes*- fase 1, realizado entre 2005 e 2006. Oslactentes foram separados em sibilantes e “não sibilantes”. Osprimeiros foram divididos de acordo com a frequência dos episódios: sibilância ocasional, quando apresentaram menos de três, e sibilância recorrente, quando manifestaram três ou mais.

**Resultados:**

A prevalência de sibilantes foi similar nas duas fases (44,6 *versus*46%). Segundo a frequência, houve aumento na prevalência de sibilância ocasional (19,4 *versus* 23%; *p*=0,03) e redução na de sibilância recorrente (26,7 *versus* 21,6%; *p*=0,005). Observou-se, ainda, aumento expressivo no diagnóstico de asma (7,5 *versus* 21,8%) e no uso de corticosteroides inalatórios (11,7 *versus* 35%), como também na hospitalização por sibilância na fase 3 (19,7 *versus* 32,6%), período da pandemia Influenza A (H1N1), o que pode ter contribuído para este desfecho.

**Conclusões:**

A prevalência da sibilância no primeiro ano de vida permanece elevada. Apesar de a avaliação temporal mostrar queda na prevalência da sibilância recorrente, aumento significante de sua morbidade foi identificado pelo maior número de hospitalizações. Além disso, houve indícios de melhora no manejo da sibilância dos lactentes, refletido pelo aumento do diagnóstico de asma e maior indicação de tratamentos preventivos.

## INTRODUCTION

Asthma is one of the most common chronic diseases in children, and its prevalence has increased in recent years.^1^ ^,^ ^2^ Most children with asthma develop symptoms in the first years of life, but diagnosing it in infants is very difficult, mainly due to the complexity in differentiating it from other frequent causes of wheezing.^3^ ^,^ ^4^

Few population studies assess the wheezing prevalence in infants, and those with a similar, standardized, and validated method, capable of allowing a comparison between different populations are even less frequent. The International Study of Wheezing in Infants *(Estudio Internacional de Sibilancias en Lactantes -* EISL) used a written and standardized questionnaire for interviews (QE-EISL - phase 1) to investigate the impact of recurrent wheezing in infants and determine its prevalence and the factors associated with it.^4^ ^,^ ^5^

The first phase of the EISL evaluated 30,093 infants. The prevalence of at least 1 episode of wheezing was 45.2%, and of recurrent wheezing (three or more episodes), 20.3%, and it was higher and more severe in Latin American than European countries.^6^ In Brazil, eight centers participated in the EISL - phase 1, one of them located in the mid-southern region of the city of São Paulo. After five years, changes in public policies related to asthma were implemented in Brazil, and new information on this topic was necessary.

The objective of this study was to determine the prevalence and severity of wheezing during the first year of life of infants who live in the mid-southern region of the city of São Paulo (EISL - phase 3) and compare these results to the data collected in the EISL - phase 1, five years after its completion.

## METHOD

Parents or guardians of infants aged 12 to 15 months, with no diseases that could affect the respiratory system, were invited to participate in interviews in which they would answer the QE-EISL - phase 3, after signing the informed consent form. The survey consisted of 50 questions about wheezing, associated respiratory symptoms, demographic characteristics, medicine consumption, and use of antibiotics and paracetamol in the first year of life. The design of the present study (EISL - phase 3) was identical to the previous one (EISL - phase 1), and used the same standardized and written questionnaire (QE-EISL - phase 3).^5^ The creators of the EISL - phase 3 recommended including at least 1,000 infants from each participating center for the sample to be significant, with an appropriate confidence level.^7^

 A single pediatrician, trained to prevent changes in the questionnaire, interviewed the participants during routine appointments or vaccination, from June 2009 to December 2010, in the mid-southern region of the city of São Paulo, similarly to the EISL - phase 1.^8^

Initially, we divided the infants into two broad categories: wheezing (at least one episode) and non-wheezing. The first group was, then, stratified according to the frequency of wheezing episodes since birth: occasional wheezing (OW) - less than three - and recurrent wheezing (RW) - three or more.

The data obtained were encoded in standard form, transferred to a database developed in Microsoft Excel, and statistically analyzed using the Statistical Package for Social Science (SPSS) for Windows, version 20.0. Categorical variables included absolute and relative frequencies; and numeric variables, summary-measures, such as mean and standard deviation. To verify the association between categorical risk factors for wheezing, we used the chi-square test and Fisher’s exact test. To compare the averages of numerical variables, we used Student’s *t*-test for independent samples. All statistical tests adopted a significance level of 5%. The Committee for Ethics in Research of Escola Paulista de Medicina, Universidade Federal de São Paulo (EPM/UNIFESP) approved this study.

## RESULTS

In phase 1, the sample comprised 1,014 infants, and 467 (46.1%) of them had at least 1 wheezing episode in the first year of life^8^. The present study - phase 3 - considered 1,335 questionnaires valid. Among them, 596 (44.6%) had at least 1 wheezing episode (p=0.496). Phase 1 identified 197 (19.4%) infants with OW,^8^ while phase 3 identified 307 (23%) (p=0.037). For RW, the results were also statistically different: phase 1 had 270 (26.6%)^8^ infants, and phase 3, 289 (21.6%) (p=0.005).

Table 1 presents the characteristics of phase 3 infants who had at least one wheezing episode, stratified by gender. The prevalence of pneumonia history and prior use of antibiotics was higher among boys. Table 2 indicates the personal and clinical characteristics of each wheezing group of phases 1 and 3 and its comparison.

**Table 1:** Clinical characteristics of infants with wheezing in the first year of life (n=596^¥^), according to gender, in the mid-southern region of the city of São Paulo - *Estudio Internacional de Sibilancias en Lactantes* - phase 3 (n=1,335^¥^).

Characteristics	Male (n=331)	Female (n=265)	Total (n=596)	OR (95%CI)	p-value^1^
	n (%)	n (%)	n (%)		
Three or more wheezing episodes	159 (48.0)	130 (49.1)	289 (48.5)	0.88 (0.50-1.45)	0.80
Six or more wheezing episodes	65 (19.6)	41 (15.5)	106 (17.8)	1.22 (0.66-1.98)	0.19
Use of inhaled B_2_ agonists	324 (97.9)	255 (96.2)	579 (97.1)	1.15 (0.76-1.87)	0.48
Use of inhaled corticosteroids	121 (36.6)	88 (33.2)	209 (35.1)	1.64 (0.32-2.44)	0.38
Use of oral antileukotrienes	27 (8.2)	22 (8.3)	49 (8.2)	0.55 (0.23-2.45)	0.84
Use of oral corticosteroids	219 (66.2)	181 (68.3)	400 (67.1)	0.60 (0.75-2.11)	0.40
Use of paracetamol in the first year of life	317 (95.8)	248 (93.6)	565 (94.8)	1.11 (0.82-1.32)	0.23
Use of antibiotics in the first year of life	289 (87.3)	209 (78.9)	498 (83.6)	*1.33 (1.09-1.88)*	0.006
Waking up at night a few times	143 (43.2)	101 (38.1)	244 (40.9)	1.19 (0.65-1.22)	0.21
Consultation in the emergency department	219 (66.2)	175 (66.0)	394 (66.1)	1.34 (0.33-1.77)	0.97
Shortness of breath noticed by parents	148 (44.7)	109 (41.1)	257 (43.1)	1.12 (0.88-1.48)	0.38
Hospitalization for wheezing	108 (32.6)	86 (32.5)	194 (32.6)	1.10 (0.61-1.21)	0.96
Asthma diagnosis	70 (21.1)	60 (22.6)	130 (21.8)	0.87 (0.73-1.34)	0.66
Pneumonia	119 (36.0)	74 (27.9)	193 (32.4)	*1.44 (1.02-1.99)*	0.037
Hospitalization for pneumonia	72 (21.8)	49 (18.5)	121 (20.3)	1.21 (0.67-1.81)	0.33

OR: Odds Ratio; 95%CI: confidence interval of 95%; ^1^chi-square test descriptive level, except for the use of inhaled B_2_ agonist (Fisher’s exact test); ^¥^596 infants showed at least one wheezing episode in a sample of 1,335 participants, which represents 44.6% of the total.

**Table 2:** Personal and clinical characteristics of infants according to wheezing episodes (occasional or recurrent) in the first year of life in the mid-southern region of the city of São Paulo. Comparison between phases 1 and 3 of the*Estudio Internacional de Sibilancias en Lactantes*.

Variables n (%)	Phase 1§		Phase 3				Phase 1§ versus phase 3			
	OW	RW	OR (95%CI)	p-value	RW	RW	OR (95%CI)	p-value	p-value	
	(n=197)	(n=270)			(n=307)	(n=289)			OW	RW
Male	100 (50.8)	159 (58.9)	1.81 (0.89-3.36)	0.08	172 (56.0)	159 (55.0)	1.18 (0.55-1.89)	0.80	0.25	0.34
Use of inhaled B_2_ agonists	162 (82.2)	240 (88.9)**	0.71 (0.12-0.97)	0.04	295 (96.1)	284 (98.3)**	1.02 (0.77-1.65)	0.15	<0.001	<0.001
Use of inhaled corticosteroids	13 (6.6)	41 (15.2)**	1.45 (1.09-1.94)	0.007	79 (25.7)	130 (45.0)**	2.33 (2.11-4.74)	<0.001	<0.001	<0.001
Use of oral antileukotrienes	4 (2.0)	8 (2.9)	1.09 (0.80-1.44)	0.70	15 (4.9)	34 (11.8)**	1.24 (1.07-1.88)	0.008	<0.001	<0.001
Use of oral corticosteroids	81 (41.1)	127 (47.0)**	0.72 (0.33-1.53)	0.20	191 (62.2)	209 (72.3)**	1.61 (1.22-2.09)	0.03	<0.001	<0.001
Waking up at night	88 (44.7)	202 (74.8)**	2.25 (1.62-3.19)	<0.001	83 (27.0)	161 (55.7)**	3.01 (2.14-3.99)	<0.001	<0.001	<0.001
Consultation in the emergency department	105 (53.3)	193 (71.5)**	2.46 (1.47-4.18)	<0.001	161 (52.4)	233 (80.6)**	1.65 (1.22-3.01)	<0.001	0.85	0.01
Shortness of breath noticed	83 (42.1)	144 (53.3)**	1.28 (1.02-1.61)	0.02	88 (28.7)	169 (58.5)**	3.43 (2.55-6.01)	<0.001	0.002	0.22
Hospitalization for wheezing	30 (15.2)	62 (23.0)**	1.13 (1.03-2.33)	0.04	75 (24.4)	119 (41.2)**	3.21 (2.51-3.88)	<0.001	0.013	<0.001
Asthma diagnosis	7 (3.5)	28 (10.4)**	1.76 (1.01-3.28)	0.007	46 (15.0)	84 (29.1)**	2.01 (1.33-3.08)	<0.001	<0.001	<0.001
Pneumonia	40 (20.3)	104 (38.5)**	3.10 (1.61-5.91)	<0.001	77 (25.1)	116 (40.1)**	3.95 (2.36-5.11)	<0.001	0.2	0.70
Hospitalization for pneumonia	25 (12.7)	46 (17.0)	1.02 (0.66-1.21)	0.20	49 (16.0)	72 (24.9)**	1.47 (1.04-1.78)	0.007	0.31	0.02

OW: occasional wheezing; RW: recurrent wheezing; OR: Odds Ratio; 95%CI: confidence interval of 95%; chi-square test descriptive p-level or Fisher’s exact test for categorical variables and Student’s *t*-test for numeric variables (weight, height, and age); **statistically significant values; §data collected from Dela Bianca et al.(2010)^8^.

The use of oral corticosteroids, the frequency of waking up at night, the need for emergency care, shortness of breath noticed by parents, and diagnosis of asthma were higher for RW than OW, in both phases of the study. However, the diagnosis of asthma (7.5 versus 21.8%) and the use of medicines to control symptoms between episodes, such as inhaled corticosteroids (11.7 versus 35.0%), increased in phase 3. Among the 130 infants diagnosed with asthma, 87 (67.0%) were treated with this class of drug.^8^

Still with respect to phase 3, in the group of wheezing infants, 20% (121/596) were hospitalized for pneumonia and 32% (194/596) for wheezing, with 91 children presenting both types of hospitalization. Hospitalization for pneumonia was more frequent among infants with RW (24.9%) than OW (16.0%) (p=0.007), unlike phase 1, which did not show statistically significant difference. Hospitalization for wheezing was more frequent in phase 3, both for infants with OW (24.4%, p=0.013) and RW (41.2%, p=0.001).

## DISCUSSION

In accordance with previous findings in the city of São Paulo, wheezing prevalence in the first year of life was very high in this study (EISL - phase 3), both for OW and RW.^8^ Results similar to those of this work - 44.6% related to at least one wheezing episode and 21.6% to RW - were found in phase 1 in other participant Brazilian centers, such as Curitiba, Porto Alegre, Recife, and Fortaleza, where 45.4, 61.0, 43.0, and 37.7% of the sample had at least one wheezing episode, and 22.6, 20.3, 24.8, and 16.2% had RW, respectively.^9^ ^,^ ^10^ ^,^ ^11^ ^,^ ^12^

Simply put, OW could be more related to the phenotype of the transient wheezing infant, with lower chances of progressing to asthma. In contrast, many studies show that RW presents good concordance with progression to asthma, especially when it happens in younger children and infants.^3^

The present study collected data five years after phase 1 and, despite the wheezing frequencies remaining high and with few statistic differences, some results warrant discussion. Thenumber of infants diagnosed with asthma (7.5 versus 21.8%) and the use of medicines to control symptoms between episodes - such as inhaled corticosteroids (11.7 versus 35%) - increased significantly in this assessment, possibly due to the interference of a few factors.

The National Plan for Asthma Control (*Plano Nacional de Controle da Asma* - PNCA), developed since 1999 by the Ministry of Health and some medical societies, enabled the public health system (*Sistema Único de Saúde* - SUS) to provide asthma medicines, and train doctors in asthma maintenance and exacerbation treatments.^13^ Medicines for mild and moderate asthma in metered dose inhaler form have been required since 2005 and became available in Basic Health Units (*Unidades Básicas de Saúde* - UBS) after this period.^14^ Thus, during the first phase of the EISL, the PNCA and the supply of asthma medicines that could be administered to children were starting, unlike when EISL- phase 3 was conducted, as the program was already consolidated by then.

It is noteworthy that asthma diagnosis in infants is difficult and must be done with caution by excluding other diseases. In this study, only 30% of infants with RW were diagnosed with asthma. In this age group, it is possible to suggest a future asthma condition for infants who have recurrent wheezing episodes and indicate a treatment similar to those administered to asthmatics, according to the current literature.^4^ Nevertheless, only 45% of infants with RW and 67% with asthma diagnosis used inhaled corticosteroids, considered the first choice of treatment. Therefore, despite the increase in RW treatment in relation to phase 1 of the EISL in São Paulo, a good part of infants was not being treated in the most effective way. Regarding the use of systemic corticosteroids for wheezing episodes, studies show controversial opinions, even in relation to RW. However, the consensus is to not use them regularly in mild cases, only in acute, more severe ones.^15^ ^,^ ^16^

Another relevant consideration that could have affected the results is the fact that, in the state of São Paulo, the term “asthma” is often replaced by “bronchitis,” both by doctors and patients, favoring a subdiagnosis of this disease.^17^

Another important result was the high prevalence of hospitalizations, especially for pneumonia. Pneumonia diagnosis in the age group under study can also be difficult and lead to confusion with other diagnoses, particularly during wheezing crises. Out of the 194 children hospitalized for wheezing, 91 were also due to pneumonia, which reinforces the imprecision of both diagnoses. However, several studies analyzed the relationship between infections and RW. Viruses have prominence with different mechanisms involved: in children with effective immune response, viruses can cause lung damage by producing free radicals, and trigger an inflammatory response by activating the nuclear factor kappa B (NF-ƘB), favoring the development of RW.^18^ On the other hand, infants who have a deficient response against viruses are more susceptible to severe and recurrent viral infections, increasing the risk of RW and asthma.^19^ It is important to emphasize that other immunological changes, including in innate response, are present in asthmatic patients, such as overproduction of mucus, which facilitates bacterial infections.^20^ Recent studies suggest that bacteria might have a solid role in the pathogenesis of asthma and that pneumonia in infants can interfere in the persistence of wheezing.^21^

The data collection period of the EISL - phase 3 coincided with the worldwide pandemic of Influenza A virus (H1N1) in 2009. According to the Notifiable Pandemic Influenza A (H1N1) Registry of the National Notifiable Diseases Information System (*Sistema Nacional de Informação de Agravos de Notificação* - SINAN), 3,278 cases reported in the state of São Paulo in this period were of children younger than 2 years. Data from the Technology Department of the Public Health System (*Departamento de Informática do Sistema Único de Saúde* - DATASUS) of the Ministry of Health shows that more than 4,000 hospitalizations of children younger than 1 year for pneumonia were recorded in the city of São Paulo from March to August 2009, while in the same period of previous years, hospitalizations did not reach 3,000.^22^ Due to the aggressiveness of the H1N1 virus, the change in routine of health professionals all over the world during the pandemic was significant.^16^ Since the disease progresses rapidly and can be deadly, the prescription of antibiotics and corticosteroids, although controversial, increased in an attempt to treat unstable cases even before confirming the virus presence. In addition, there was a high demand for emergency services by the general population.^23^ ^,^ ^24^ ^,^ ^25^ ^,^ ^26^ In this regard, the H1N1 pandemic can be considered a bias in the results of this research and justify the great frequency of use of antibiotics and the increase in administration of oral corticosteroids compared to phase 1.

The use of a questionnaire that depended on the memory of parents and/or guardians can also lead to information bias and be a limitation of this study. However, some studies reveal that parents can remember their children’s diseases with precision, particularly regarding recent facts as those occurred in the previous year.^27^ Epidemiological studies that use a questionnaire are extremely effective and low-cost in producing information and improvements in public policies. It is an instrument of great value, especially for developing countries.

Another point was the wheezing theme. Parents or guardians of infants might confuse wheezing and other respiratory sounds, resulting in doubts and, consequently, a mistaken estimate of its actual prevalence. However, to validate the construction of the EISL questionnaire, a summarized version identified good concordance between the perception of parents and medical diagnosis after auscultation.^28^

In conclusion, the wheezing prevalence in the first year of life remains significant and with high morbidity in the mid-southern region of the city of São Paulo. Nonetheless, despite the temporal assessment showing a decrease in the prevalence of RW, a significant increase in its morbidity was identified due to the higher number of hospitalizations and consumption of specific medicines for its treatment.

Changes in current public policies, such as facilitating the access of infants with RW to specialized services and specific training on childhood asthma, could improve the outlook of this condition in São Paulo.
